# Diversity of *Tilletiopsis*-Like Fungi in Exobasidiomycetes (Ustilaginomycotina) and Description of Six Novel Species

**DOI:** 10.3389/fmicb.2019.02544

**Published:** 2019-11-22

**Authors:** Christian Richter, Andrey M. Yurkov, Teun Boekhout, Marc Stadler

**Affiliations:** ^1^Department of Microbial Drugs, Helmholtz Centre for Infection Research, Braunschweig, Germany; ^2^German Collection of Microorganisms and Cell Cultures, Leibniz Institute DSMZ, Braunschweig, Germany; ^3^Yeast Division, Westerdijk Fungal Biodiversity Institute, Utrecht, Netherlands; ^4^Institute for Biodiversity and Ecosystem Dynamics, University of Amsterdam, Amsterdam, Netherlands

**Keywords:** six new species, *Tilletiopsis*, apple, white haze, postharvest disorder

## Abstract

In 2006 several yeast-like fungi were isolated from apples that showed a postharvest disorder named “white haze.” These strains were morphologically and molecularly assigned to the genus *Tilletiopsis*. Following the recent reclassification of yeasts in Ustilaginomycotina and the genus *Tilletiopsis* in particular, species that caused “white haze” disorder were re-identified based on the phylogenetic analysis of five DNA-loci (ITS, LSU, SSU, *RPB2*, and *TEF1*) and analysis of D1/D2 domains of the 26S/28S rRNA (LSU). Six novel species belonging to three orders in the Exobasidiomycetes, namely *Entyloma belangeri* (holotype: CBS 111600; ex-type: DSM 29114) MB 823155, *Entyloma davenportii* (holotype: CBS 111604; ex-type: DSM 100135) MB 823154, *Entyloma elstari* (holotype: CBS 111593; ex-type: DSM 29113) MB 823153, *Entyloma randwijkense* (holotype: CBS 111606; ex-type: DSM 100136) MB 823156, *Jamesdicksonia mali* (holotype: CBS 111625; ex-type: DSM 29121) MB 823151 and *Golubevia heteromorpha* (holotype: CBS 111610; ex-type: DSM 100176) MB 823152 are proposed to accommodate these strains. In addition, sequences representing phylogenetically related but yet undescribed fungi were obtained from GenBank in order to show the diversity of *Tilletiopsis*-like yeast states in Exobasidiomycetes.

## Introduction

Species of the genus *Tilletiopsis* are saprotrophic yeast-like basidiomycete fungi. The name *Tilletiopsis* was first introduced by Derx (1930) and was chosen to reflect the morphological resemblance of his isolates to species of the smut fungi *Tilletia*. Eighteen years later, Derx (1948) described the genus without designating a type species (Derx, 1948). Unfortunately, the strain he was working with got lost so that Nyland (1950) selected a morphologically similar strain to serve as the neotype of the type species of the genus *Tilletiopsis*, which he named *Tilletiopsis washingtonensis*. Another species with smaller ballistospores was named *Tilletiopsis minor*, and both yeasts were isolated near Puyallup (Washington, United States) from living and dead plant material (Nyland, 1950). Two years later Tubaki described two additional species and a variety based on spore size and culture morphology, namely *Tilletiopsis cremea*, *Tilletiopsis lilacina*, and *T. minor* var. *flava* (Tubaki, 1952). In 1972 Gokhale added *Tilletiopsis fulvescens*, *Tilletiopsis albescens* and *Tilletiopsis pallescens* to the genus (Gokhale, 1972). In 1991 the name *Tilletiopsis flava* was proposed for *T. minor* var. *flava* (Boekhout, 1991). The latest described species were *Tilletiopsis derxii*, *Tilletiopsis oryzicola*, and *Tilletiopsis penniseti* (Takashima and Nakase, 2001). The treatment of *T. cremea* and *T. lilacina* as synonyms of *T. washingtonensis* (Boekhout, 1998) was disproved by molecular studies of Hamamoto et al. (2000).

Traditionally, phenotypic, i.e., morphological and physiological, criteria were used to distinguish yeast species. In culture, yeasts of the genus *Tilletiopsis* are distinguished by tough to soft colonies that are cream, pinkish-cream, pinkish-yellow, yellow-brown or brown in color, with a reticulate, transversely wrinkled, postulate or velutinous surface (Boekhout, 1998, 2011). The micromorphology of species is rather uniform including narrow, septate, hyaline, monokaryotic regularly branched hyphae with retraction septa, and cytoplasm-containing cells that are usually surrounded by lysed cells (Boekhout, 1998, 2011). Ballistoconidia occur in species of *Tilletiopsis* (Boekhout, 1998, 2011). With the development of identification methods (reviewed in Barnett, 2004; Kurtzman et al., 2011) biochemical characteristics such as Q-10 as major ubiquinone, and cell walls without xylose, positive Diazonium blue B and urease reactions, and the inability to produce starch-like compounds were found to be characteristic for the genus (Boekhout, 1991, 2011; Boekhout et al., 1992). As has been noted by several authors, the morphology of *Tilletiopsis* spp. resembles that of some smut fungi, such as *Entyloma* and *Tilletia*, suggesting possible anamorph-teleomorph relationships between these yeasts and plant parasites in Exobasidiomycetes (e.g., Boekhout, 1987, 1991). Application of ribosomal gene sequencing confirmed the phylogenetic relationships of the genus *Tilletiopsis* in Exobasidiomycetes, but also indicated that the genus *Tilletiopsis* is polyphyletic (Boekhout et al., 1995; Takashima and Nakase, 1996). Further studies confirmed that species of *Tilletiopsis* are placed in three orders, namely Georgefischerales, Entylomalates, and Doassansiales, and some species represent the anamorphic stage of known teleomorphic species (Begerow et al., 2000; Fell et al., 2000; Bauer et al., 2001, 2006; Boekhout, 2011; Wang et al., 2015).

Recent changes introduced by the International Code of Nomenclature for algae, fungi, and plants (ICN, Melbourne Code, 2012; McNeill et al., 2012) discontinued the use of dual nomenclature for naming fungi with a pleomorphic life-cycle and the so-called “One Fungus = One Name” principle (Hawksworth, 2011; Taylor, 2011) was applied to unify nomenclature of sexual and asexual fungi in the phylum Ustilaginomycotina (Wang et al., 2015). In the revision of the genus *Tilletiopsis* by Wang et al. (2015) most of the described species were reclassified in either new genera (i.e., *Robbauera* and *Golubevia*) or already existing teleomorphic genera (i.e., *Phragmotaenium* and *Gjaerumia*). Specifically, *T. albescens* was recombined as *Robbauera albescens*; *T. pallescens* as *Golubevia pallescens*; *T. derxii*, *T. flava*, *T. fulvescens*, and *T. oryzicola* as *Phragmotaenium derxii*, *Phragmotaenium flavum*, *Phragmotaenium fulvescens*, and *Phragmotaenium oryzicola*, respectively; *T. penniseti* and *T. minor* as *Gjaerumia penneseti* and *Gjaerumia minor*, respectively. Only the type species, *T. washingtonensis*, and the two related species *T. cremea* and *T. lilacina* remained in the genus *Tilletiopsis*. Despite previously supposed close relationships with the genus *Entyloma*, none of the described *Tilletiopsis* species was transferred to this genus.

Species of the genus *Tilletiopsis sensu lato* are ubiquitous and can be found in various environments, and the most frequently reported habitats of these yeasts include diverse, either dead or living, plant material (Boekhout, 1991). Additionally, the propagules have been detected in the air, from where these yeasts can be transferred to flowers, plant surfaces, soils, sewage and deep-sea sediments, seawater and even sea animals (Gokhale, 1972; Shivas and Brown, 1989; Fairs et al., 2010; Li et al., 2011; Mahé et al., 2014; Yue et al., 2015). *T. minor* is the only known species of this genus, that was reported from clinical specimens and is a possible causing agent of pneumonia and corneal abscess (Ramani et al., 1997; Kechkekian et al., 2007; AL-Zaydani et al., 2014). Some species may have evolved a mycoparasitic lifestyle as has been suggested for *T. pallescens* and *T. albescens* (Boekhout, 2011; Begerow et al., 2014). Interestingly, these *Tilletiopsis* species show potential as biocontrol agents against powdery mildews, especially against the Cucurbitaceae pathogens of *Podosphaera* (Last, 1970; Kiss, 2003). The active principle seems to be a fatty acid ester, and similar compounds are already known to be secreted by other members of the Ustilaginomycotina, including the commercially available biocontrol agent *Pseudozyma flocculosa* (Urquhart and Punja, 2002), which was erroneously reclassified in the genus *Anthracocystis* due to the errors with the type material of this species (Richard R. Bélanger and T. Boekhout, personal communication).

Together with other conventional epiphytes, *Tilletiopsis* occurs on the surface of apples where its growth matters as a disruptive element in the commercial production and distribution of the fruit (Boekhout et al., 2006). Different species were isolated from apples in several European countries and were ultimately linked to the postharvest disorder named “white haze,” an intensive fungal growth on the apple fruit surface which results in a compromised quality of the fruits (Boekhout et al., 2006; Baric et al., 2010; Weber and Zabel, 2011; Prencipe et al., 2016). A high relative humidity and lower temperatures were suggested as key factors controlling the disorder that appears after Ultra-Low Oxygen storage (Boekhout et al., 2006).

Several potential new species of the genus *Tilletiopsis* were discovered from apples showing white haze disorder (Boekhout et al., 2006). The identification based on the rDNA sequencing (D1/D2 domains of the LSU and ITS) together with morphological analyses suggested their close relationships with the genus *Entyloma* and other genera within the Exobasidiomycetes (Boekhout et al., 2006). Because these cultures were not included in the study by Wang et al. (2015) and because the genus *Tilletiopsis* is currently restricted to the clade containing its generic type *T. washingtonensis*, we performed a multi-locus phylogenetic analysis to provide a proper placement of these hitherto undescribed species. In addition to the nucleotide sequences determined by Boekhout et al. (2006), we analyzed partial sequences of the ribosomal small subunit (SSU rDNA), as well as fragments of the genes encoding the second largest subunit of RNA polymerase II (*RPB2*) and the translation elongation factor 1 alpha (*TEF1*). Herein we describe six new species and indicate possible novel taxa based on the phylogenetic analysis of LSU rDNA sequences from public repositories.

## Materials and Methods

All 28 studied strains were isolated by Boekhout et al. (2006) and preserved in the Westerdijk Fungal Biodiversity Institute, Utrecht, Netherlands (Table 1). Temperature growth tests were performed on MEA, GPYA, and PDA. Physiological tests were performed in liquid culture at 10°C and examined after 1, 2, 3, and 4 weeks. Nucleotide sequences of ITS and LSU of those strains have been published before by Boekhout et al. (2006). Other LSU sequences obtained from NCBI GenBank^1^ and analyzed here represent potential novel species that were originally identified as members of the genus *Tilletiopsis* by the authors (Fungsin, 2003; Jindamorakot, 2006; Takashima et al., 2012). Reference sequences were derived from GenBank and from Wang et al. (2015). All sequence and strain accession numbers for the five-loci analysis are listed in Table 2. Clades and strains of the new species are labeled (A–G) after the classification of Boekhout et al. (2006).

**TABLE 1:** Strains and GenBank accession numbers of new species, proposed type strains in bold.

**Strain**	**Proposed name**	**Clade**	**ITS**	**LSU**	**SSU**	**RPB2**	**TEF1**
**CBS 111625**	*Jamesdicksonia mali*	A	AY879279	AY879274	LT615037	LT614983	LT615009
CBS 111628	*Jamesdicksonia mali*	A	AY879281	AY272007	LT615038	LT614984	LT615010
**CBS 111610**	*Golubevia heteromorpha*	B	AY259058	AY272003	LT615026	LT614973	LT614999
CBS 111608	*Golubevia heteromorpha*	B	AY259056	AY272001	LT615024	LT614971	LT614997
CBS 111609	*Golubevia heteromorpha*	B	AY259065	AY272011	LT615025	LT614972	LT614998
CBS 111611	*Golubevia heteromorpha*	B	AY259060	AY272005	LT615027	LT614974	LT615000
CBS 111612	*Golubevia heteromorpha*	B	AY879275	AY879270	LT615028	LT614975	LT615001
CBS 111614	*Golubevia heteromorpha*	B	AY259079	AY272032	LT615029	LT614976	LT615002
CBS 111615	*Golubevia heteromorpha*	B	AY259053	AY272025	LT615030	LT614977	LT615003
CBS 111616	*Golubevia heteromorpha*	B	AY259049	AY272022	LT615031	LT614978	LT615004
CBS 111617	*Golubevia heteromorpha*	B	AY259050	AY272023	LT615032	LT614979	LT615005
CBS 111618	*Golubevia heteromorpha*	B	AY259078	AY272031	LT615033	LT614980	–
CBS 111620	*Golubevia heteromorpha*	B	AY879276	AY879273	LT615034	LT614981	LT615006
CBS 111621	*Golubevia heteromorpha*	B	AY259082	AY272035	LT615035	LT614982	LT615007
CBS 111741	*Golubevia heteromorpha*	B	AY259061	AY272006	LT615036	–	LT615008
**CBS 111604**	*Entyloma davenportii*	E	AY259064	AY272010	LT615013	LT614961	LT614987
CBS 111607	*Entyloma davenportii*	C	AY259051	AY272024	LT615011	LT614959	LT614985
CBS 111603	*Entyloma davenportii*	E	AY259054	AY272026	LT615012	LT614960	LT614986
**CBS 111593**	*Entyloma elstari*	D	AY259048	AY272021	LT615023	DQ234552	DQ028593
**CBS 111600**	*Entyloma belangeri*	F	AY259074	AY272019	LT615019	LT614967	LT614993
CBS 111596	*Entyloma belangeri*	F	AY259055	AY272027	LT615015	LT614963	LT614989
CBS 111597	*Entyloma belangeri*	F	AY259070	AY272015	LT615016	LT614964	LT614990
CBS 111598	*Entyloma belangeri*	F	AY259071	AY272016	LT615017	LT614965	LT614991
CBS 111599	*Entyloma belangeri*	F	AY259075	AY272020	LT615018	LT614966	LT614992
CBS 111601	*Entyloma belangeri*	F	AY259072	AY272017	LT615020	LT614968	LT614994
CBS 111602	*Entyloma belangeri*	F	AY259069	AY272028	LT615021	LT614969	LT614995
CBS 111605	*Entyloma belangeri^∗^*	G1	AY259076	AY272029	LT615022	LT614970	LT614996
**CBS 111606**	*Entyloma randwijkense*	G2	AY259080	AY272033	LT615014	LT614962	LT614988

*^∗^Provisionally assigned to *Entyloma belangeri* species complex.*

**TABLE 2:** Species, strains, and GenBank accession numbers for the five-loci tree.

**Species**	**LSU**	**ITS**	**SSU**	**RPB2**	**TEF1**
*Entyloma arnoseridis* CBS 203.36^∗^	DQ645528	DQ911609	DQ645529	DQ645530	DQ645531
*Entyloma calendulae* CBS 513.93	DQ663687	DQ663689	DQ663688	DQ663690	DQ663691
*Entyloma ficariae* CBS 480.91^∗^	AJ235295	–	KP322949	KP323102	KP323125
*Exobasidium gracile* DSM 4460^∗^	DQ663699	DQ663700	DQ785786	DQ663701	DQ663703
*Gjaerumia minor* CBS 543.50^T*^	AJ235287	KP322989	KP322972	KP323097	KP323114
*Gjaerumia penniseti* CBS 110032^T*^	AB052825	–	KP322975	KP323085	KP323143
*Golubevia pallescens* CBS 364.85^T*^	AJ235292	–	KP322973	KP323101	KP323123
*Microstroma juglandis* CBS 287.63^∗^	AF009867	DQ789988	DQ789987	DQ789989	DQ789991
*Phragmotaenium derxii* CBS 110078^T*^	AB052823	AB045707	AB045704	KP323086	KP323138
*Phragmotaenium flavum* CBS 401.84^T*^	AJ235285	KP322987	KP322970	–	KP323126
*Phragmotaenium fulvescens* CBS 607.83^T*^	AJ235282	KP322988	KP322971	KF706530	KF706483
*Rhamphospora nymphaeae* CBS 172.38^∗^	DQ831032	DQ831034	DQ831033	DQ831035	DQ831036
*Robbauera albescens* CBS 608.83^T*^	AJ235289	KP322986	KP322968	KP323095	KP323127
*Tilletia goloskokovii* AFTOL-ID 1713^∗^	AY818998	DQ832248	DQ832247	DQ832249	DQ832251
*Tilletiaria anomala* CBS 436.72^T*^	AJ235284	DQ234558	AY803752	AY803750	DQ835991
*Tilletiopsis cremea* CBS 605.83^T*^	AJ235279	AB025690	KP322969	KP323108	KP323129
*Tilletiopsis lilacina* CBS 435.92^T*^	AJ235309	KP322984	KP322966	KP323110	KP323112
*Tilletiopsis washingtonensis* CBS 544.50^T*^	AJ235278	DQ835994	KP322976	DQ835995	DQ835996
*Mycosarcoma maydis* CBS 504.76^∗^	AF453938	AY854090	KP322979	KP323090	KP323130

*^*T*^Sequences from type material; ^∗^sequences used by Wang et al. (2015).*

Genomic DNA was extracted from cultures grown on potato dextrose agar (PDA, BD Difco), using the EZ-10 Spin Column Genomic DNA kit for plant samples (Bio Basic Canada Inc., Markham, Canada). The extractions were performed using the manufacturer’s protocol. For cell disruption a Precellys 24 homogenizer (Bertin Technologies, France) was used at a speed of 6000 rpm for 2 × 40 s. Four gene regions were amplified, namely a part of the ribosomal small subunit (SSU or 18S rRNA gene), the internal transcribed spacer region (ITS) and fragments of the two protein-coding genes encoding the second largest RNA polymerase II subunit (*RPB2*) and the translation elongation factor 1 alpha (*TEF1*). Primers ITS1 and ITS4 (White et al., 1990), NS23UCB and NS24UCB (Gargas and Taylor, 1992) were used to amplify SSU, RPB2-6F and RPB2-7R primers to amplify *RPB2* (Liu et al., 1999), and EF1-983F and EF1-2218R to amplify *TEF1* (Matheny et al., 2007). All primer sequences are provided at the PriMicro Database Project^2^ (Supplementary Table S1). PCR products were purified with the EZ-10 Spin Column PCR Purification kit (Bio Basic Canada Inc., Markham, Canada) and the FastGene Gel/PCR Extraction kit (Nippon Genetics Europe GmbH, Düren, Germany) following the manufacture’s protocols. Purified PCR amplification products were sequenced by the department of Genome Analytics at the Helmholtz Centre for Infection Research (Braunschweig, Germany), using the same primers as used for the PCR reaction.

Independent alignments and phylogenetic analyses were performed for each locus. Multiple sequence alignments were performed with the nucleotide sequences using MAFFT 7.017 algorithm (Katoh et al., 2002) with default parameters. The resulting alignments were additionally cured with Gblocks (Castresana, 2000; Talavera and Castresana, 2007) allowing smaller final blocks, gap positions within the final blocks, and less strict flanking positions. The following two datasets were used in the phylogenetic analyses: (i) a five-loci dataset (ITS, LSU, SSU, *RPB2*, and *TEF1*) was used to analyse the placement of the novel species within the Exobasidiomycetes and (ii) a LSU dataset was used to analyse nucleotide sequences of potential novel species available in public databases. Single-gene alignments corresponding to five-loci were concatenated and the best nucleotide substitution model was determined with MEGA 7.0.14 (Kumar et al., 2016). Phylogenetic relationships were inferred by the maximum likelihood (ML) method based on the general time reversible (GTR + G + I) model for the five-loci dataset and K80 + G + I for the LSU dataset, respectively. Trees were calculated with RAxML 7.2.8 (Stamatakis, 2006) and the PhyML (Guindon and Gascuel, 2003) plugins implemented in Geneious 7.1.4 (Biomatters Ltd., Auckland, New Zealand), followed by 1000 bootstrap replicates. *Mycosarcoma* (formerly *Ustilago*) *maydis* was selected as an outgroup.

## Results

**FIGURE 1:**
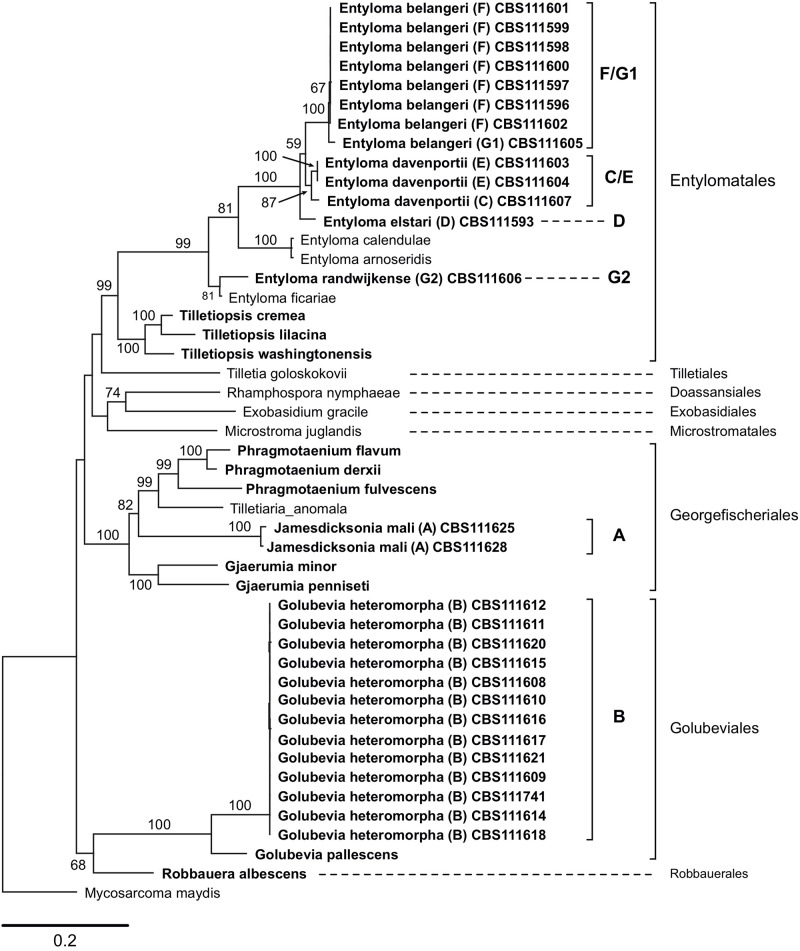
Maximum likelihood analysis of a five-loci alignment combining sequences of the ITS region (including 5.8S rRNA gene), LSU (D1/D2 domains), and SSU rRNA gene, *RPB2*, and *TEF1*, showing the relationships of taxa within the Exobasidiomycetes. The numbers on branches are frequencies (>50%) with which a given branch appeared in 1000 bootstrap replications. The scale bars indicate the numbers of expected substitutions accumulated per site. Nucleotide sequences of *Tilletiopsis*-like fungi are in bold; clade classification (A–G) follows (Boekhout et al., 2006).

**FIGURE 2:**
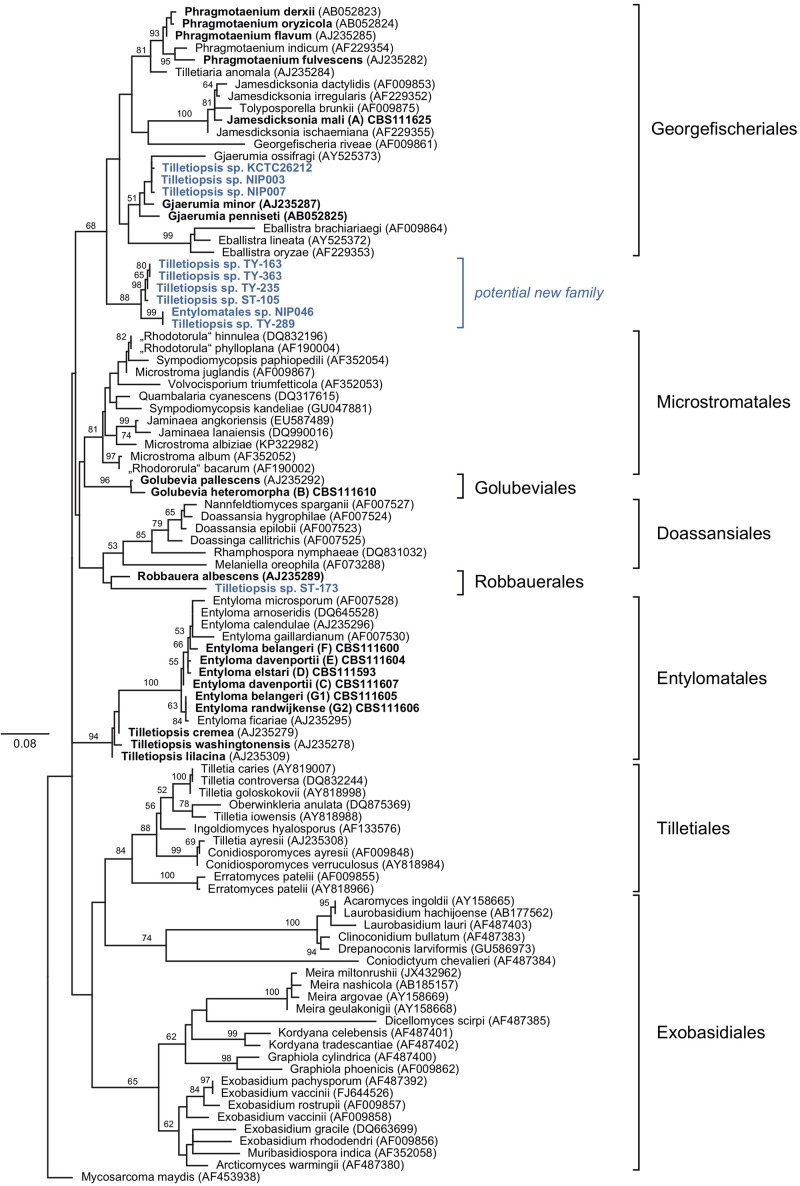
Diversity of *Tilletiopsis*-like fungi determined with Maximum Likelihood analysis of the LSU rRNA gene (D1/D2 domains). The numbers on branches are frequencies (>50%) with which a given branch appeared in 1000 bootstrap replications. The scale bars indicate the numbers of expected substitutions accumulated per site. Nucleotide sequences of *Tilletiopsis*-like fungi are in bold. Potential new species are given in blue color.

Sequences that were generated in this study, and those obtained from GenBank, reference sequences from Wang et al. (2015) and data published by Boekhout et al. (2006) were used to produce two phylogenetic trees (Figures 1, 2). The five-loci tree provides an overview on the phylogenetic relationships of asexual and sexual taxa in Exobasidiomycetes (Figure 1). The orders Tilletiales, Doassansiales, Microstromatales, and Exobasidiales were represented by a single species to provide a better overview and not to overload the tree. In a taxonomically broad analysis based on LSU rRNA sequences (Figure 2), new species described in this study were represented by a few sequences only. In both analyses, the placement of six new species was resolved. Specifically, they were placed in orders Georgefischeriales, Golubeviales, and Entylomatales. Both trees showed, however, a rather weak support for higher ranks, but the respective families received a good support.

In the genus *Entyloma*, pairwise sequence comparisons showed 90–98% similarity in the ITS region and 98–100% similarity in LSU. Intraspecific variation of ITS sequences in *Entyloma davenportii* was 3%. Most strains of *Entyloma belangeri* showed highly similar ITS (99–100% similarity) and LSU (100% similarity) sequences but nucleotide sequences of strain CBS 111605 showed only 92% similarity in ITS and 98% similarity in LSU. Two strains of *Jamesdicksonia mali* shared identical LSU sequences, whereas ITS sequences showed 94% similarity. In the genus *Tilletiopsis*, interspecific pairwise sequence comparisons resulted in 99 and 92–95% similarity values for LSU and ITS sequences, respectively.

In the five-loci phylogenetic analysis, two strains (clade A in Boekhout et al., 2006) were placed in the Georgefischeriales in an intermediate position between the already known *Tilletiopsis* species, which were recently transferred to the genera *Gjaerumia* and *Phragmotaenium* (Wang et al., 2015). This result was also supported by the LSU analysis, where the clade A was placed in the genus *Jamesdicksonia* with high support (ML: 100%). Clade B represented by 13 strains was placed in the order Golubeviales close to the recently reclassified *G. pallescens* with high support (ML: 100 and 96% in five-loci and LSU analyses, respectively). The other four new species were placed in the order Entylomatales (clades C/E, F/G1, D, and G2) close to *Entyloma ficariae* (Figure 1). Despite some observed sequence heterogeneity between strains (9% ITS and 2% LSU), our results suggest that clade F/G1 are likely to represent one new species (ML: 100%). The two other clades C/E and D (one strains) constitute two other novel species (Figure 1). The total intraspecific sequence similarity within clades F/G1 and C/E was 98% (Supplementary Data Sheet S1). The LSU tree supported the placement of these species in Entylomatales, but also showed two conflicts between the trees, namely (i) the placement of strain G1 next to G2 in close relationship to *E. ficariae*, and (ii) the separation of the clade C/E in the LSU tree (Figure 2). However, the five-loci tree strongly supported the placement of new species within the order Entylomatales, while internal nodes in Entylomatales received no support in the LSU analysis (Figures 1, 2). As several smut and false smut fungi, species of the genus *Entyloma* are distinguished based on the combination of morphology (e.g., spore measurements), host plant, and genetic information, usually ITS sequences (e.g., Begerow et al., 2002; Boekhout et al., 2006; Lutz and Pia̧tek, 2016). Because plant parasites have been studied by traditional mycologists and phytopathologists, only a few *Entyloma* species are available in culture and three of them have been used by Wang et al. (2015) in the multi-gene phylogenetic analysis. The lack of cultures and nucleotide sequence data (e.g., sequences of protein-coding genes) from teleomorphic species is hampering a better taxonomic placement in many fungal lineages (Kachalkin et al., 2019), including smuts (e.g., Wang et al., 2015; Begerow et al., 2017). Right now it is impossible to univocally interpret the unexpectedly high variability of ITS sequences among *E. belangeri* isolates. This result contradicts to some extent current views on species delimitation in the genus *Entyloma* (e.g., Lutz and Pia̧tek, 2016), but it is important to document that total sequence variability was low (Supplementary Data Sheet S1). In the lack of host data and teleomorph morphology, phylogenetic characters, i.e., results of the multi-gene analysis, are used here to delimit new species. We are in favor of a rather conservative decision to keep strain CBS 111605 provisionally assigned to *E. belangeri* species complex until more *Entyloma* specimens and cultures will be sequenced.

In addition to the afore-mentioned new species, sequences obtained from public databases were included in the LSU dataset to provide a broader look on the genetic diversity of fungi morphologically resembling the genus *Tilletiopsis*. These nucleotide sequences represent potential undescribed species. Our analysis placed a few of these sequences in two orders of the Exobasidiomycetes, namely Georgefischeriales and Robbauerales, and in a new clade that represents a distinct cluster close to the order Georgefischeriales (Figure 2). The sequence of ST-173 (GenBank: DQ404470) was placed close to *R. albescens*, the only presently known species in the order Robbauerales. Despite low statistical support, this sequence represents most likely the second species in this order. Three sequences retrieved from GenBank (AB726595, AB726598, AF459717) were placed in the Georgefischeriales close to *G. penneseti* and *G. minor*, but with little statistical support in the analysis. Sequences of these strains display high pair-wise similarity and likely represent a new species. Six strains formed a clade close to Georgefischeriales (Figure 2). The clade itself received moderate support (ML: 88%), but its relationship with the order Georgefischeriales was not supported (ML: 68%). Our analysis suggests that this new clade likely corresponds to a new family or order. Within the clade sequences NIP046 (AB726628) and TY-289 (AY313022) probably represent one well-supported (ML: 99%) new species, and the sister clade with four more strains (DQ404458, AY313021, AY313023, AY313020) could represent at least one more new species.

From an ecological point of view, it is important to note that the species studied here were involved in a post-harvest disorder on apples, called white haze (Boekhout et al., 2006) that occurred most prominently in the apple harvest season during humid and colder periods and under Low Oxygen storage. As standard growth tests of yeasts are done at 25°C our first attempts were also done at this temperature. However, several of the strains did not grow well or not at all at this temperature. Therefore, we investigated the growth range of the isolates at temperatures ranging from 4 to 30°C on three different media. All isolates tested grew well at 4–15°C, and hence we repeated the growth tests, both carbon and nitrogen compounds, at 10°C. All studied cultures required a longer period of ca. 2 weeks to start growing well, but once growth started quite some visible biomass in the test tubes was accumulated.

## Taxonomy

### Description of *Jamesdicksonia mali* Richter, Yurkov, and Boekhout, sp. nov. (MB 823151)

Etymology: The specific epithet *mali* refers to the plant genus *Malus* Mill. (Rosaceae), which includes apple trees, the source of isolation.

**FIGURE 3:**
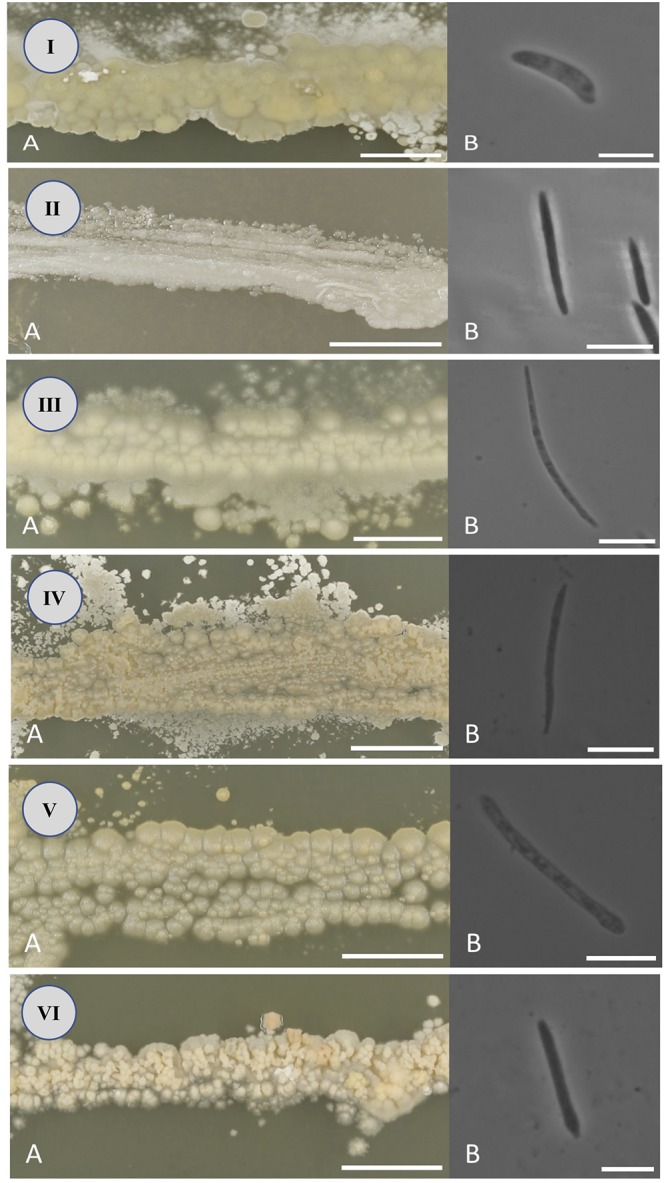
Colony **(A)** and ballistospore **(B)** morphology of *Jamesdicksonia mali* **(I)**, *Golubevia heteromorpha* **(II)**, *Entyloma elstari* **(III)**, *Entyloma davenportii* **(IV)**, *Entyloma belangeri* **(V)**, and *Entyloma randwijkense* **(VI)** on potato dextrose agar (bars: **A** = 10 mm, **B** = 10 μm).

After 2 weeks at 22°C on PDA, the colonies are tough, wrinkled, dull, covered with slender fascicles, yellow to cream colored, with an entire to slightly ridged margin. Ballistospores are allantoid or cylindrical, 2–3 × 12–19 μm (Figure 3I).

Fermentation of glucose absent. Growth on glucose, galactose, D-glucosamine (at 10°C), D-ribose, D-xylose (delayed), L-arabinose, rhamnose (weak or negative), sucrose, maltose, trehalose, cellobiose, arbutin (weak), melibiose, lactose, raffinose, melezitose, inuline, soluble starch, glycerol (weak), erythritol (weak), arabinitol (delayed), glucitol, mannitol, D-gluconate, glucoronate, succinate, and quinic acid. No growth on sorbose, D-arabinose, methyl-alpha-D-glucoside, salicin, ribitol, xylitol, galactitol, inositol, glucono-delta-lactone, 2-keto-gluconate, D-galacturonate, saccharate, lactate, citrate, methanol, ethanol, propane-1,2-diol, butane-2,3-diol, and galactonic acid. Growth on potassium nitrate, sodium nitrite, lysine, and ethylamine (weak or negative). No growth on cadaverine, creatinine, creatinine, D-glucosamine and imidazole. No growth in the presence of 0.01% cycloheximide, in the presence of 10% NaCl and in 50% glucose. Urea hydrolysis and Diazonium Blue B reaction are positive. Optimal growth temperature: 15–22°C. Maximum growth temperature: 25°C; growth at 30°C is weak (on MEA) or negative (on GPYA).

Molecular characteristics (holotype): Nucleotide sequences of ITS, SSU, LSU (D1/D2 domains) rRNA and *RPB2* and *TEF1* deposited in NCBI/EMBL (GenBank) under the accession numbers AY879279 (ITS), AY879274 (LSU), LT615037 (SSU), LT614983 (*RPB2*), LT615009 (*TEF1*).

Deposits: Holotype CBS 111625 isolated from an apple of the cultivar Elstar showing signs of white haze obtained through the Plant Protection Service [Plantenziektenkundige Dienst (PD)] in Wageningen, Netherlands in 1994 (Boekhout et al., 2006), preserved in a metabolically inactive state in the CBS collection of the Westerdijk Fungal Biodiversity Institute, Utrecht, Netherlands. Ex-type culture is deposited at the German Collection of Microorganisms and Cell Cultures, Braunschweig, Germany (DSM 29121).

Strains studied: paratypes CBS 111625 (=DSM 29121), CBS 111628 (=DSM 29122).

### Description of *Golubevia heteromorpha* Boekhout, Richter, and Yurkov, sp. nov. (MB 823152)

Etymology: The specific epithet *heteromorpha* (Gr. adj. f., *hetero –* different, *morphe* shape) refers to a diverse morphological appearance as plate culture on culture medium.

After 2 weeks at 22°C on PDA, the colonies are tough, wrinkled, shiny or dull, covered with slender fascicles, cream colored or whitish, with an entire to slightly ridged margin. Ballistospores are cylindrical, elongate, or lunate, 1–2 × 12–20 μm (Figure 3II).

Fermentation of glucose absent. Growth on glucose, sorbose (sometimes delayed), L-arabinose (sometimes weak), D-arabinose, sucrose, maltose, trehalose, melibiose (delayed), raffinose (delayed), melezitose (delayed), inulin (weak), and soluble starch (delayed). Variable growth on galactose, ribose, xylose, methyl-alpha-D-glucoside, cellobiose, arbutin, lactose, glycerol, erythritol, ribitol, xylitol, arabinitol, glucitol, mannitol, inositol, glucono-delta-lactone, 2-keto-gluconate, succinate, and quinic acid. No growth on D-glucosamine, rhamnose, salicin, galactitol, gluconate, glucoronate, galacturonate, saccharate, lactate, citrate, methanol, ethanol, propane-1,2-diol, butane-2,3-diol, and galactonic acid. Growth on potassium nitrate, sodium nitrite, lysine (variable) and imidazole (variable). No growth on ethylamine, cadaverine, creatine, creatinine, and D-glucosamine. No growth in the presence of 0.01% cycloheximide, in the presence of 10% NaCl and in 50% glucose. Urea hydrolysis and Diazonium Blue B reaction are positive. Optimal growth temperature: 15–22°C. Maximum growth temperature: 20°C; some strains can grow at 25°C on MEA.

Molecular characteristics (holotype): Nucleotide sequences of ITS, SSU, LSU (D1/D2 domains) rRNA and *RPB2* and *TEF1* deposited in NCBI/EMBL (GenBank) under the accession numbers AY259058 (ITS), AY272003 (LSU), LT615026 (SSU), LT614973 (*RPB2*), LT614999 (*TEF1*).

Deposits: Holotype CBS 111610 isolated from an apple of the cultivar Elstar showing signs of white haze obtained through the Plant Protection Service [Plantenziektenkundige Dienst (PD)] in Wageningen, Netherlands in 1994 (Boekhout et al., 2006), preserved in a metabolically inactive state in the CBS collection of the Westerdijk Fungal Biodiversity Institute, Utrecht, Netherlands. Ex-type culture is deposited at the German Collection of Microorganisms and Cell Cultures, Braunschweig, Germany (DSM 100176).

Strains studied: paratypes CBS 111610 (=DSM 100176), CBS 111608 (=DSM 100175), CBS 111609 (=DSM 29125), CBS 111611 (=DSM 100138), CBS 111612 (=DSM 100177), CBS 111614 (=DSM 100139), CBS 111615 (=DSM 100140), CBS 111616 (=DSM 100141), CBS 111617 (=DSM 100142), CBS 111618 (=DSM 100143), CBS 111620 (=DSM 100144), CBS 111621 (=DSM 100145), CBS 111741 (=DSM 29124).

### Description of *Entyloma elstari* Yurkov, Boekhout, and Richter, sp. nov. (MB 823153)

Etymology: The specific epithet *elstari* refers to the apple cultivar Elstar, which is the source of isolation.

After 2 weeks at 22°C on PDA, the colonies are tough, slightly wrinkled, dull, pale whitish-tan colored, with a ridged margin. Ballistospores are elongate, falcate or lunate, 0.5–2 × 16–32 μm (Figure 3III).

Fermentation of glucose absent. Growth on glucose, galactose (weak), ribose (delayed), xylose (latent), L-arabinose (delayed), sucrose, maltose, trehalose, methyl-alpha-D-glucoside (latent), cellobiose (delayed), melibiose (latent), lactose (latent), raffinose, melezitose, inulin (weak), soluble starch (weak), glycerol (delayed), erythritol (latent), ribitol (latent), xylitol (latent), arabinitol (latent), glucitol (weak), mannitol (delayed), and inositol (latent). No growth on sorbose, D-glucosamine, D-arabinose, rhamnose, salicin, arbutin, galactitol, 2-keto-gluconate, gluconate, glucoronate, galacturonate, saccharate, lactate, succinate, citrate, methanol, ethanol, propane-1,2-diol, butane-2,3-diol, quinic acid, and galactonic acid. Growth on potassium nitrate and sodium nitrite. No growth on lysine, ethylamine, cadaverine, creatine, creatinine, D-glucosamine, and imidazole. No growth in the presence of 0.01% cycloheximide, in the presence of 10% NaCl and in 50% glucose. Urea hydrolysis and Diazonium Blue B reaction are positive. Optimal growth temperature: 15–22°C. Maximum growth temperature: 25°C; growth at 30°C is weak (on MEA) or negative (on GPYA).

Molecular characteristics (holotype): Nucleotide sequences of ITS, SSU, LSU (D1/D2 domains) rRNA and *RPB2* and *TEF1* deposited in NCBI/EMBL (GenBank) under the accession numbers AY259048 (ITS), AY272021 (LSU), LT615023 (SSU), DQ234552 (*RPB2*), DQ028593 (*TEF1*).

Deposits: Holotype CBS 111593 isolated from an apple of the cultivar “Elstar” showing signs of “white haze” was obtained from an Ultra-Low Oxygen storage room in Netherlands in 1998 (Boekhout et al., 2006), preserved in a metabolically inactive state in the CBS collection of the Westerdijk Fungal Biodiversity Institute, Utrecht, Netherlands. An ex-type culture is deposited at the German Collection of Microorganisms and Cell Cultures, Braunschweig, Germany (DSM 29113).

Strain studied: CBS 111593 (=DSM 29113).

### Description of *Entyloma davenportii* Yurkov, Richter, and Boekhout, sp. nov. (MB 823154)

Etymology: The specific epithet *davenportii* is dedicated to R. R. Davenport, in recognition of his contribution to his ecological studies of yeasts on fruits.

After 2 weeks at 22°C on PDA, the colonies are soft, wrinkled or furrowed, dull, whitish-cream colored, with a ridged margin. Ballistospores are elongate, falcate or lunate, 1–2 × 13–24 μm (Figure 3IV).

Fermentation of glucose absent. Growth on glucose, galactose, ribose (sometimes weak), xylose (sometimes weak), L-arabinose, sucrose, trehalose, cellobiose, raffinose, inulin (delayed), melezitose, soluble starch, glycerol (delayed), erythritol, arabinitol (weak), and mannitol (delayed).

No growth on sorbose, D-glucosamine, D-arabinose, rhamnose, maltose, methyl-alpha-D-glucoside, salicin, arbutin, melibiose, lactose, ribitol, xylitol, glucitol, galactitol, inositol, 2-keto-gluconate, gluconate, glucoronate, galacturonate, saccharate, lactate, succinate, citrate, methanol, ethanol, propane-1,2-diol, butane-2,3-diol, quinic acid, and galactonic acid. Growth on potassium nitrate, sodium nitrite, lysine, and creatinine. No growth on ethylamine, cadaverine, creatine, D-glucosamine, and imidazole. No growth in the presence of 0.01% cycloheximide, in the presence of 10% NaCl and in 50% glucose. Urea hydrolysis and Diazonium Blue B reaction are positive. Optimal growth temperature: 15–22°C. Maximum growth temperature: 25°C.

Molecular characteristics (holotype): Nucleotide sequences of ITS, SSU, LSU (D1/D2 domains) rRNA and *RPB2* and *TEF1* deposited in NCBI/EMBL (GenBank) under the accession numbers AY259064 (ITS), AY272010 (LSU), LT615013 (SSU), LT614961 (*RPB2*), LT614987 (*TEF1*).

Deposits: Holotype CBS 111604 isolated from an apple of the cultivar “Elstar” showing signs of “white haze” obtained through the Plant Protection Service [Plantenziektenkundige Dienst (PD)] in Wageningen, Netherlands in 1994 (Boekhout et al., 2006), preserved in a metabolically inactive state in the CBS collection of the Westerdijk Fungal Biodiversity Institute, Utrecht, Netherlands. An ex-type culture is deposited at the German Collection of Microorganisms and Cell Cultures, Braunschweig, Germany (DSM 100135).

Strains studied: paratypes CBS 111604 (=DSM 100135), CBS 111603 (=DSM 100134), CBS 111607 (=DSM 100137).

### Description of *Entyloma belangeri* Boekhout, Richter, and Yurkov, sp. nov. (MB 823155)

Etymology: The specific epithet *belangeri* is dedicated to Richard R. Bélanger, who developed Sporodex^®^, a commercial powdery mildew biocontrol agent, based on *P. flocculosa*.

After 2 weeks at 22°C on PDA, the colonies are tough, slightly wrinkled, dull, white or cream colored, with a slightly ridged margin. Ballistospores are elongate, cylindrical or allantoid, 1–2 × 18–29 μm (Figure 3V).

Fermentation of glucose absent. Growth on glucose, galactose, ribose (weak), xylose (variable), L-arabinose, sucrose, maltose (variable), trehalose, cellobiose (variable), raffinose, melezitose (variable), inulin (weak, delayed), soluble starch (weak), glycerol (weak), erythritol (weak), ribitol (variable), and mannitol (weak). No growth on sorbose, D-glucosamine, D-arabinose, rhamnose, methyl-alpha-D-glucoside, salicin, arbutin, melibiose, lactose, xylitol, arabinitol, glucitol, galactitol, inositol, glucono-delta-lactone, 2-keto-gluconate, gluconate, glucoronate, galacturonate, saccharate, lactate, succinate, citrate, methanol, ethanol, propane-1,2-diol, butane-2,3-diol, quinic acid, and galactonic acid. Growth on potassium nitrate, sodium nitrite, and imidazole (variable). No growth on lysine, ethylamine, cadaverine, creatine, creatinine, and D-glucosamine. No growth in the presence of 0.01% cycloheximide, in the presence of 10% NaCl and in 50% glucose. Urea hydrolysis and Diazonium Blue B reaction are positive. Optimal growth temperature: 15–22°C. Maximum growth temperature: 25°C growth at 30°C is variable.

Molecular characteristics (holotype): Nucleotide sequences of ITS, SSU, LSU (D1/D2 domains) rRNA and *RPB2* and *TEF1* deposited in NCBI/EMBL (GenBank) under the accession numbers AY259074 (ITS), AY272019 (LSU), LT615019 (SSU), LT614967 (*RPB2*), LT614993 (*TEF1*).

Deposits: Holotype CBS 111600 isolated from an apple of the cultivar “Elstar” showing signs of russetting obtained from the orchard Krabbendijk, Netherlands in 1998 (Boekhout et al., 2006), preserved in a metabolically inactive state in the CBS collection of the Westerdijk Fungal Biodiversity Institute, Utrecht, Netherlands. An ex-type culture is deposited at the German Collection of Microorganisms and Cell Cultures, Braunschweig, Germany (DSM 29114).

Strains studied: CBS 111600 (=DSM 29114), CBS 111596 (=DSM 100129), CBS 111597 (=DSM 100130), CBS 111598 (=DSM 100131), CBS 111599 (=DSM 100132), CBS 111601 (=DSM 100133), CBS 111602 (=DSM 29115).

Note: strain CBS 111605 (=DSM 29116) has been provisionally assigned to *E. belangeri* species complex based on results of the multi-gene phylogenetic analysis (Figure 1). Nucleotide sequences of this strain showed 98 and 92% similarity to the type culture in LSU and ITS regions, respectively.

### Description of *Entyloma randwijkense* Richter, Boekhout, and Yurkov, sp. nov. (MB 823156)

Etymology: The specific epithet *randwijkense* refers to the Dutch village Randwijk, which was the location of the experimental station from which the isolation source (apples) derived.

After 2 weeks at 22°C on PDA, the colonies are tough, flat, furrowed, dull, creamish-tan colored, with an entire margin. Ballistospores are cylindric, allantoid or falcate, 1–2 × 8–22 μm (Figure 3VI).

Fermentation of glucose absent. Growth on glucose, galactose (sometimes weak), L-arabinose (delayed), rhamnose, sucrose, maltose (weak), trehalose, cellobiose (weak), raffinose, melezitose (delayed), inulin (weak), soluble starch (weak), glycerol (latent), erythritol (latent), mannitol (weak), and 2-keto-gluconate (latent).

No growth on sorbose, D-glucosamine, ribose, xylose, D-arabinose, methyl-alpha-D-glucoside, salicin, arbutin, melibiose, lactose, ribitol, xylitol, arabinitol, glucitol, galactitol, inositol, glucono-delta-lactone, gluconate, glucoronate, galacturonate, saccharate, lactate, succinate, citrate, methanol, ethanol propane-1,2-diol, butane-2,3-diol, quinic acid, and galactonic acid. Growth on potassium nitrate (weak), sodium nitrite (weak), lysine (weak), and imidazole. No growth on ethylamine, cadaverine, creatinine, creatinine, and D-glucosamine. No growth in the presence of 0.01% cycloheximide, in the presence of 10% NaCl and in 50% glucose. Urea hydrolysis and Diazonium Blue B reaction are positive. Optimal growth temperature: 15–22°C. Maximum growth temperature: 25°C.

Molecular characteristics (holotype strain): Nucleotide sequences of ITS, SSU, LSU (D1/D2 domains) rRNA and *RPB2* and *TEF1* deposited in NCBI/EMBL (GenBank) under the accession numbers AY259080 (ITS), AY272033 (LSU), LT615014 (SSU), LT614962 (*RPB2*), LT614988 (*TEF1*).

Deposits: Holotype CBS 111606 isolated from an apple obtained from the orchard Wognum II, Netherlands in 1998 (Boekhout et al., 2006), preserved in a metabolically inactive state in the CBS collection of the Westerdijk Fungal Biodiversity Institute, Utrecht, Netherlands. An ex-type culture is deposited at the German Collection of Microorganisms and Cell Cultures, Braunschweig, Germany (DSM 100136).

Strain studied: CBS 111606 (=DSM 100136).

### Correction of *Tilletiopsis albescens* Gokhale, Nova Hedwigia 23: 801 (1972) (MB 324630)

The species *T. albescens* was described by Gokhale (1972) based on a culture isolated by Cooke from sewage in Libertyville, IL, United States. The type material (culture UBC 926) was clearly designated by the author but the material was deposited not in a single but in two institutions, i.e., in Mycology Herbarium of the University of British Columbia (UBC) and in the American Type Culture Collection (ATCC). However, according to the catalog of the UBC herbarium, the specimen UBC F926 is *Lycogala epidendrum*, a species of slime mold (accessed on June 6, 2019). The original culture from UBC herbarium was deposited in the CBS culture collection (presently the Westerdijk Fungal Biodiversity Institute) by Bandoni. This culture is permanently preserved in a metabolically inactive state under the number CBS 608.83. In accordance with the Art. 9.2 of the ICN (Shenzhen code, 2017; Turland et al., 2018) we modify the protologue as following:

Deposits: original culture UBC 926 isolated from filamentous organisms from sewage in Libertyville, IL, United States (Gokhale, 1972) was deposited as two isotypes preserved in a metabolically inactive state in the CBS collection of the Westerdijk Fungal Biodiversity Institute (Utrecht, Netherlands) as CBS 608.83 and in the American Type Culture Collection (Manassas, United States) as ATCC 24343.

Recently, Wang et al. (2015) recombined *T. albescens* as *R. albescens* and designated this species as the type species of the genus *Robbauera*.

### Correction of *Tilletiopsis pallescens* Gokhale (MB 324632) and Validation of Golubevia Q. M. Wang, F. Y. Bai, Begerow, and Boekhout (MB 812694) and *Golubevia pallescens* (Gokhale) Q. M. Wang, F. Y. Bai, Begerow, and Boekhout (MB 812695)

The species *T. pallescens* was described by Gokhale (1972) based on a culture isolated from a fruiting body of *Sirobasidium* sp. collected in Shamoda, Japan. The type material (culture UBC 8007) was clearly designated by the author but the material was deposited not in a single but in two institutions, i.e., in Mycology Herbarium of the University of British Columbia (UBC) and in the American Type Culture Collection (ATCC). However, according to the catalog of the UBC herbarium, the specimen UBC F8007 is *Hebeloma glutinosum*, a basidiomycete (accessed on June 6, 2019). The original culture from UBC herbarium was deposited in the CBS culture collection (presently the Westerdijk Fungal Biodiversity Institute) by Bandoni. This culture is permanently preserved in a metabolically inactive state under the number CBS 606.83. In accordance with the Art. 9.2 of the ICN (Shenzhen code, 2017; Turland et al., 2018) we modify the protologue as following:

Deposits: original culture UBC 8007 isolated from a fruiting body of *Sirobasidium* sp. collected in Shamoda, Japan (Gokhale, 1972) was deposited as two isotypes preserved in a metabolically inactive state in the CBS collection of the Westerdijk Fungal Biodiversity Institute (Utrecht, Netherlands) as CBS 608.83 and in the American Type Culture Collection (Manassas, United States) as ATCC 24345.

Recently, Wang et al. (2015) recombined *T. pallescens* as *G. pallescens*. However, the basionym *T. pallescens* was not indicated in the publication. Therefore, this taxonomic combination is invalid according to Art. 41.5 of the ICN (Shenzhen code, 2017; Turland et al., 2018). Here, we provide validation of *G. pallescens*, the type species of the genus *Golubevia*.

*Golubevia pallescens* (Gokhale) Q. M. Wang, F. Y. Bai, Begerow and Boekhout comb. nov. Studies in Mycology 81: 78 (2015) (MB 812695).

Basionym: *T. pallescens* Gokhale, Nova Hedwigia 23: 805 (1972) (MB 324632).

*Golubevia* Q. M. Wang, F. Y. Bai, Begerow and Boekhout, Studies in Mycology 81: 78 (2015); MycoBank MB 812694.

Type species: *G. pallescens* (Gokhale) Q. M. Wang, F. Y. Bai, Begerow and Boekhout, Studies in Mycology 81: 78 (2015); MycoBank MB 812695.

The genus was proposed by Wang et al. (2015) to accommodate a single species *T. pallescens*. The second species of the genus is described in the present study as *G. heteromorpha* Boekhout, Richter and Yurkov (MB 823152).

## Discussion

In this study, we analyze and formally describe six new species belonging to three orders of the Exobasidiomycetes. These species were resolved in the multigene tree based on five DNA-loci, and in the LSU-based phylogenetic tree. In addition, our analyses included sequences that represent potential new species (Figures 1, 2). Comparing the results from the two analyses, we suggest that it is generally important to enlarge a phylogenetic analysis with additional DNA-loci. As an example, strain G1, which was placed close to G2 in the LSU dataset, clustered with the clade F when four additional loci were included in the analysis. Additional protein-coding gene fragments improved separation of strains and statistical support of the clades, as compared to the LSU-based tree. However, even this five-loci dataset was not sufficient to resolve relationships between the orders in Exobasidiomycetes, as displayed by low bootstrap values.

Asexual yeast and yeast-like states of plant parasites in Ustilaginomycotina are already known for almost 100 years (reviewed in Begerow et al., 2014, 2017). Some of them produce anamorphs *in situ* (e.g., *Ustilago*, *Entyloma*, *Anthracoidea*, *Exobasidium*), but the characteristics of these asexual morphs have been neglected from the taxonomy of this group as it was solely based on the characters describing sexual reproduction. Fungi morphologically similar to the anamorphs of Ustilaginomycotina were frequently isolated from sources other than the known host plants, for example soil, air or animal samples (e.g., Boekhout, 2011; Begerow et al., 2014, 2017). Also isolation of such yeasts from healthy and non-host plants were repeatedly reported (Inácio et al., 2008; Begerow et al., 2014, 2017). For a long time these yeasts were described in often large asexual genera like *Candida*, *Rhodotorula*, *Sporotrichum*, and *Sterigmatomyces*, until a rather stable system of dual naming of sexual and asexual members of Ustilaginomycotina was established (Boekhout, 1998, 2011; Bauer et al., 2001; Begerow et al., 2014, 2017). Most of the yeasts were accommodated in the two genera *Pseudozyma* (anamorphic Ustilaginaceae) and *Tilletiopsis* (anamorphic Exobasidiomycetes). Because the nomenclature of plant parasites and yeasts developed independently, big formal genera spanning over taxonomic families and orders were long a workable solution. With the reclassification of teleomorphic species based on cell ultrastructure and DNA-markers (e.g., Bauer et al., 2001), it became obvious that the genera comprising asexual morphs are also polyphyletic (e.g., Fell et al., 2000; Scorzetti et al., 2002) and should be split in the future (e.g., Inácio et al., 2008). After the use of dual nomenclature for the taxonomy of fungi with pleomorphic life cycle was terminated from 2013 (Hawksworth, 2011; Taylor, 2011), it became necessary to come up with a new concept and to revise sexual and asexual taxa together.

The former concept of the genus *Tilletiopsis*, which was originally based on morphological characteristics, was changing and expanding along with a growing number of species discovered. However, it was still based on the characteristics of the type species (*T. washingtonensis*), so this genus could not suit a place to accommodate other asexual Exobasidiomycetes, such as *Jaminaea* (Sipiczki and Kajdacsi, 2009), *Sympodiomycopsis* (Sugiyama et al., 1991), and *Quambalaria* (Simpson, 2000). With the introduction of ribosomal DNA sequencing techniques, the genus *Tilletiopsis* turned out to be polyphyletic, containing species of different orders scattered across the Exobasidiomycetes. This situation was similar to another large anamorphic genus, *Pseudozyma*, which also served as a “catch-it-all”-genus in the Ustilaginomycetes (Bauer et al., 2001; Boekhout, 1998, 2011; Begerow et al., 2014, 2017). With time, the concept of the genus *Tilletiopsis* to accommodate all yeast states in Exobasidiomycetes was changed and, consequently, novel genera circumscribed with molecular phylogenetic data were erected, e.g., *Jaminaea*, *Acaromyces* (Boekhout, 2003; Sipiczki and Kajdacsi, 2009). These genera may also represent anamorphic stages of plant parasites, as suggested by the recent phylogenetic analyses (Wang et al., 2015), but the lack of nucleotide sequence data from teleomorphic species is hampering a better taxonomic placement. Phylogenetic analyses performed by Wang et al. (2015) and in this study show that the genus *Tilletiopsis* was polyphyletic. Also, sequences from undescribed species derived from public databases suggest that additional phylogenetic clades comprising *Tilletiopsis*-like yeasts are to be expected. Some of them might represent a new family or order in the Exobasidiomycetes close to Georgefischeriales. However, phylogenetic placement of these species would require a solid multi-gene analysis in the future.

Asexual yeast states in Exobasidiomycetes are known to produce compounds which are active against other organisms (reviewed in Begerow et al., 2014). Antagonistic reactions toward other fungi were reported for members of genera *Acaromyces*, *Golubevia*, *Meira*, *Moesziomyces*, *Tilletiopsis*, and *Sympodiomycopsis* (reviewed in Begerow et al., 2014). Similarly to a few formerly classified in the genus *Tilletiopsis* species (e.g., Urquhart and Punja, 2002; Kiss, 2003; Begerow et al., 2014), antagonistic interactions of *Golubevia heteromorpha* (cited as *T. pallescens*) against powdery mildew *Blumeria graminis* f. sp. *tritici* has been recently reported (Köhl et al., 2019). This yeast proved to be a promising biocontrol agent which significantly reduced the number of powdery mildew pustules (Köhl et al., 2019).

## Data Availability Statement

The datasets generated for this study can be accessed from GenBank, provided in Tables 1, 2.

## Author Contributions

CR, TB, and AY performed the experiments. TB, MS, and AY designed the experiments. CR, TB, AY, and MS wrote the manuscript.

## Conflict of Interest

The authors declare that the research was conducted in the absence of any commercial or financial relationships that could be construed as a potential conflict of interest.

## Footnotes

1 http://www.ncbi.nlm.nih.gov/genbank

2 http://primicro.jpn.org
