# Supplementary material for: Diversity of Tilletiopsis-Like Fungi in Exobasidiomycetes (Ustilaginomycotina) and Description of Six Novel Species
Source: Front Microbiol. 2019 Nov 22;10:2544. doi: 10.3389/fmicb.2019.02544 (PMC6883903; doi:10.3389/fmicb.2019.02544)
Supplement: Supplementary file 1 [file Table_1.docx]

**Table S1 |** Primers used in this study.

| **Primer** | **Sequence** | **Reference** |
| --- | --- | --- |
| ITS1 | TCCGTAGGTGAACCTGCGG | White et al., 1990 |
| ITS4 | TCCTCCGCTTATTGATATGC | White et al., 1990 |
| NS23UCB | GACTCAACACGGGGAAACTC | Gargas and Taylor, 1992 |
| NS24UCB | AAACCTTGTTACGACTTTTA | Gargas and Taylor, 1992 |
| RPB2-6F | TGGGGKWTGGTYTGYCCTGC | Liu et al., 1999 |
| RPB2-7R | CCCATWGCYTGCTTMCCCAT | Li et al., 2008 |
| EF1-983F | GCYCCYGGHCAYCGTGAYTTYAT | Matheny et al., 2007 |
| EF1-2218R | ATGACACCRACRGCRACRGTYTG | Matheny et al., 2007 |
