# Supplementary material for: Diversity of Tilletiopsis-Like Fungi in Exobasidiomycetes (Ustilaginomycotina) and Description of Six Novel Species
Source: Front Microbiol. 2019 Nov 22;10:2544. doi: 10.3389/fmicb.2019.02544 (PMC6883903; doi:10.3389/fmicb.2019.02544)
Supplement: Supplementary file 2 [file Data_Sheet_1.docx]

**Supplementary DataSheet 1: Additional phylogenetic analyses**

Singe-gene phylogenetic trees were inferred by the maximum likelihood (ML) method based on the general time reversible (GTR) model with RaxML (version 7.2.8) using raxmlGUI 1.5 and the GTRCAT option with 100 rounds of bootstrap replicates (Silvestro and Michalak, 2012). In order to test for possible incongruences while combining data from the analyzed gene loci, single-gene ML trees were combined into single file and analyzed with Splitstree 4.10 using the ConsensusNetwork algorithm (Huson and Bryant, 2006).

In the concatenated five-loci Maximum Likelihood phylogenetic tree made with (Fig. 1) and without CBS 111605 (Fig. S1), the overall topology and support for clades did not change suggesting no major conflicts between single-gene datasets. In the consensus network (Fig. S2), ML trees were based on alignments of 12 taxa representing clades C, D, E, F, and G1 (Fig. 1), and containing 526 bp of ITS, 467 bp of LSU, 740 of SSU, 638 bp of *RPB2* and 642 bp of *TEF1*. Clade F/G1 was resolved in ITS (ML: 99%), SSU (ML: 100%), *RPB2* (ML: 100%) and *TEF1* (ML: 100%) single-gene trees. Due to overall low sequence heterogeneity, LSU tree resolved only E and F clades. This result is also consistent with results of the phylogenetic analysis depicted in Fig. 2. Two consensus phylogenetic networks were calculated to visualize contrasting results of pairwise sequence similarity values of ITS and total intraspecific sequence similarity within clades F/G1 and C/E (see Results). The network analyses revealed intraspecific heterogeneity of *Entyloma belangeri* and *Entyloma davenportii* (Fig. S2), as also suggested by the pairwise sequence similarity values (see Results). Those analyses confirmed the separation of the two novel species (Fig. S2). The analysis depicted in Fig. S1 also suggests that, despite its separate position in ITS phylogenetic tree (data not shown), strain CBS 111605 should rather be included in *Entyloma belangeri* than considered as a separate species. We are in favor of a rather conservative decision to keep strain CBS 111605 provisionally assigned to *Entyloma belangeri* species complex until more *Entyloma* specimens and cultures will be sequenced.

**References**

Silvestro D, Michalak I (2012). raxmlGUI: a graphical front-end for RAxML. *Org. Divers. Evol.* 12, 335-337. doi: 10.1007/s13127-011-0056-0

Huson DH, Bryant D (2006). Application of phylogenetic networks in evolutionary studies. *Mol. Biol. Evol.* 23, 254-267. doi: 10.1093/molbev/msj030

**Figure S1 |** Maximum Likelihood analysis of a five-loci alignment combining sequences of the ITS region (including 5.8S rRNA gene), LSU (D1/D2 domains) and SSU rRNA gene, *RPB2*, and *TEF1*, showing the relationships of taxa within the Exobasidiomycetes. The numbers on branches are frequencies (>70 %) with which a given branch appeared in 100 bootstrap replications. The scale bars indicate the numbers of expected substitutions accumulated per site. Nucleotide sequences of *Tilletiopsis*-like fungi are in bold; clade classification (A-G) follows Boekhhout et al. (2006).


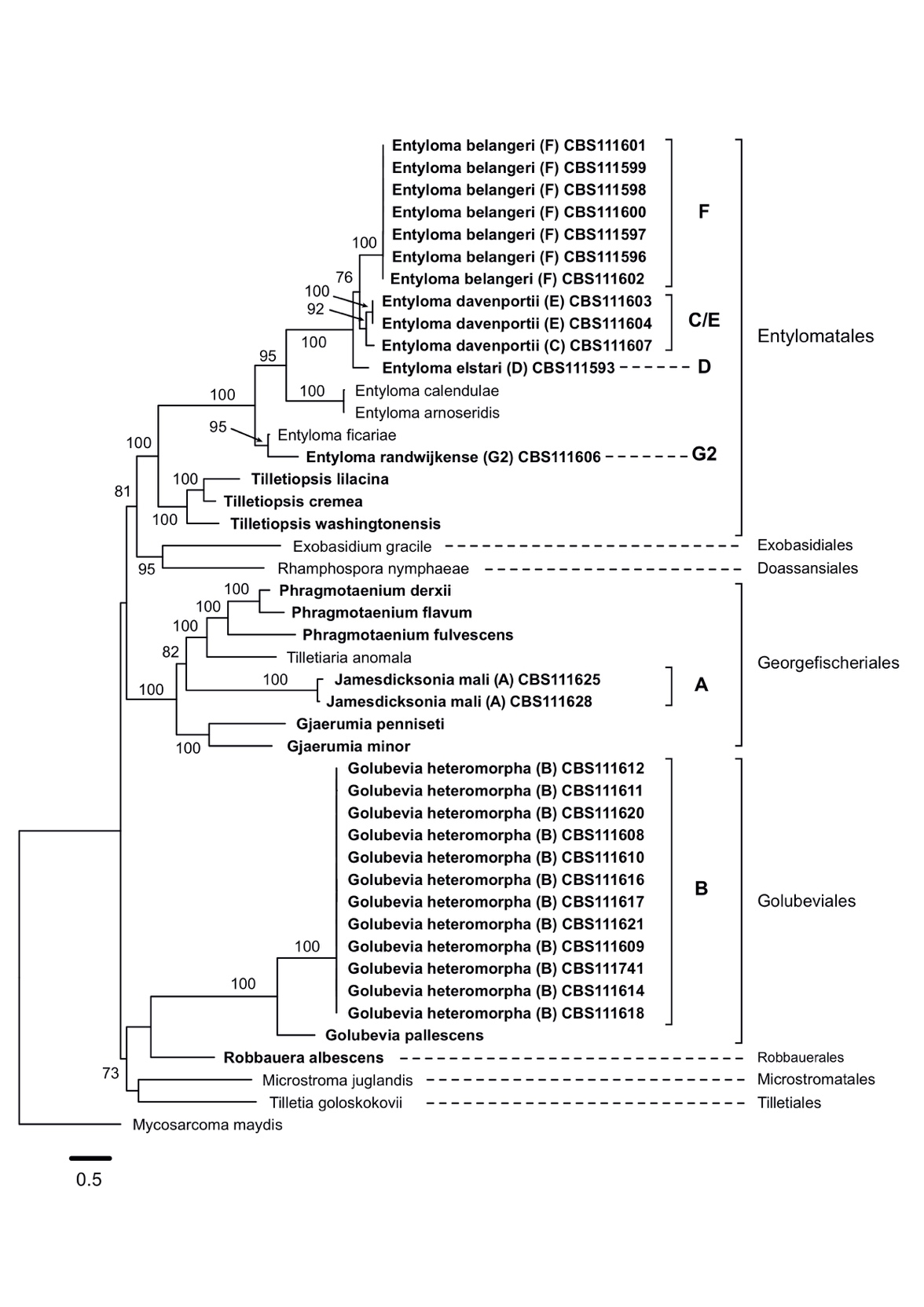


**Figure S2 |** Consensus network analyses.

**Single-gene trees used for consensus network analyses**

**LSU Maximum Likelihood tree**

best_ml_lsu=((((111597:0.00000130385933774295,111598:0.00000130385933774295):0.00000130385933774295,(111602:0.00000130385933774295,(111596:0.00000130385933774295,((111599:0.00000130385933774295,111600:0.00000130385933774295):0.00000130385933774295,111601:0.00000130385933774295):0.00000130385933774295):0.00000130385933774295):0.00000130385933774295):0.00865485792397793080,111607:0.00000130385933774295):0.00000130385933774295,((111604:0.00000130385933774295,111603:0.00000130385933774295):0.00215026321693151260,111605:0.00647712643053639358):0.00000130385933774295,111593:0.00215415811917043978):0.0;

**SSU Maximum Likelihood tree**

best_ml_ssu=((111607:0.00000078842353933909,(111604:0.00000078842353933909,111603:0.00000078842353933909):0.02810920140805165529):0.01222013879711842874,((((111597:0.00000078842353933909,((111602:0.00000078842353933909,111605:0.00000078842353933909):0.00000078842353933909,(111598:0.00000078842353933909,111600:0.00000078842353933909):0.00000078842353933909):0.00000078842353933909):0.00000078842353933909,111599:0.00000078842353933909):0.00000078842353933909,111601:0.00000078842353933909):0.00000078842353933909,111596:0.00000078842353933909):0.02203885704787500802,111593:0.02442243138730133128):0.0;

**ITS Maximum Likelihood tree**

best_ml_its=((111604:0.00000087861578783429,111603:0.00000087861578783429):0.00214966021897016043,(111607:0.01999185719265793998,(((((111597:0.00000087861578783429,111601:0.00000087861578783429):0.00000087861578783429,111600:0.00000087861578783429):0.00000087861578783429,(111596:0.00000087861578783429,111602:0.00000087861578783429):0.00000087861578783429):0.00000087861578783429,(111599:0.00000087861578783429,111598:0.00000087861578783429):0.00000087861578783429):0.00811648048040906379,111605:0.04506921912124076807):0.01558960330470265943):0.00275818812272499419,111593:0.00818794340602229005):0.0;

**TEF1 Maximum Likelihood tree**

best_ml_tef=(111607:0.00113439090123562724,((111604:0.00000133934189537085,111603:0.00000133934189537085):0.00064667507770136291,(111602:0.00000133934189537085,((((111605:0.00000133934189537085,111601:0.00000133934189537085):0.00000133934189537085,111596:0.00000133934189537085):0.00000133934189537085,111598:0.00000133934189537085):0.00000133934189537085,(111599:0.00000133934189537085,(111600:0.00000133934189537085,111597:0.00000133934189537085):0.00000133934189537085):0.00000133934189537085):0.00263802993704660444):0.03628354397954452332):0.00487063692848998218,111593:0.02408701704260565735):0.0;

**RPB2 Maximum Likelihood tree**

best_ml_rpb=((111607:0.02469772668757795608,(111604:0.00000111975165730850,111603:0.00000111975165730850):0.00979491903961730859):0.01566180706174570211,(((111596:0.00000111975165730850,111597:0.00000111975165730850):0.00000111975165730850,111602:0.00000111975165730850):0.00000111975165730850,((111605:0.00000111975165730850,111601:0.00000111975165730850):0.00000111975165730850,(111600:0.00000111975165730850,(111599:0.00000111975165730850,111598:0.00000111975165730850):0.00000111975165730850):0.00000111975165730850):0.00000111975165730850):0.03078141682504497920,111593:0.06340644965759260898):0.0;
